# Novel Rodent Models for Macular Research

**DOI:** 10.1371/journal.pone.0013403

**Published:** 2010-10-15

**Authors:** Gesine Huber, Severin Heynen, Coni Imsand, Franziska vom Hagen, Regine Muehlfriedel, Naoyuki Tanimoto, Yuxi Feng, Hans-Peter Hammes, Christian Grimm, Leo Peichl, Mathias W. Seeliger, Susanne C. Beck

**Affiliations:** 1 Division of Ocular Neurodegeneration, Centre for Ophthalmology, Institute for Ophthalmic Research, University of Tuebingen, Tuebingen, Germany; 2 Laboratory of Retinal Cell Biology, Department of Ophthalmology, University of Zurich, Zurich, Switzerland; 3 5th Medical Department, Universitätsmedizin Mannheim, University of Heidelberg, Mannheim, Germany; 4 Max Planck Institute for Brain Research, Frankfurt am Main, Germany; University of Florida, United States of America

## Abstract

**Background:**

Many disabling human retinal disorders involve the central retina, particularly the macula. However, the commonly used rodent models in research, mouse and rat, do not possess a macula. The purpose of this study was to identify small laboratory rodents with a significant central region as potential new models for macular research.

**Methodology/Principal Findings:**

*Gerbillus perpallidus*, *Meriones unguiculatus* and *Phodopus campbelli*, laboratory rodents less commonly used in retinal research, were subjected to confocal scanning laser ophthalmoscopy (cSLO), fluorescein and indocyanine green angiography, and spectral-domain optical coherence tomography (SD-OCT) using standard equipment (Heidelberg Engineering HRA1 and Spectralis™) adapted to small rodent eyes. The existence of a visual streak-like pattern was assessed on the basis of vascular topography, retinal thickness, and the topography of retinal ganglion cells and cone photoreceptors. All three species examined showed evidence of a significant horizontal streak-like specialization. cSLO angiography and retinal wholemounts revealed that superficial retinal blood vessels typically ramify and narrow into a sparse capillary net at the border of the respective area located dorsal to the optic nerve. Similar to the macular region, there was an absence of larger blood vessels in the streak region. Furthermore, the thickness of the photoreceptor layer and the population density of neurons in the ganglion cell layer were markedly increased in the visual streak region.

**Conclusions/Significance:**

The retinal specializations of *Gerbillus perpallidus*, *Meriones unguiculatus* and *Phodopus campbelli* resemble features of the primate macula. Hence, the rodents reported here may serve to study aspects of macular development and diseases like age-related macular degeneration and diabetic macular edema, and the preclinical assessment of therapeutic strategies.

## Introduction

**Figure 1:**
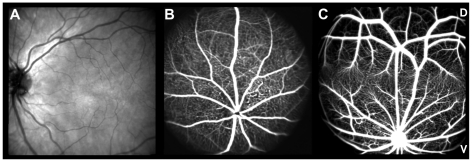
*In vivo* fundus imaging. cSLO fundus images of (**A**) human, (**B**) mouse, and (**C**) Meriones unguiculatus. (**A–C**) D = dorsal, V = ventral. (**A**) Native infrared (λ = 830 nm) SLO image of one researcher's right eye illustrates the sparse vascularization of the macular region. (**B**) Fluorescein angiography (FLA) (488 nm, barrier filter at 500 nm) reveals the evenly distributed retinal blood vessels in mice. (**C**) FLA image of Meriones unguiculatus visualizes the horizontal sparse vascular band denoting the specialized retinal region dorsal to the optic disc.

The retina is the neurosensory tissue within the eye that allows us to access the visual environment. It has a layered architecture designed for optimal light perception in rod and cone photoreceptors and subsequent processing of the visual information in second and third order neurons. Further, non-neuronal cells (blood vessels and glial cells) serve the functional and structural integrity of this tissue. Presumably to optimize the visual information about the environment, topographical differences within the retina have developed in several species. Vision in humans and other primates heavily depends on an area of very high resolution, the macula (Fig. 1A), which is characterized by a greatly enhanced density of cones, cone bipolar cells, and ganglion cells.

Diseases that specifically involve the macula, so-called maculopathies, are thus particularly disabling for the affected patients. A prominent example is age-related macular degeneration (AMD), a group of diseases that constitutes the most common cause of untreatable blindness in the Western world. AMD is regularly a slowly progressing process and mainly affects people over age 60 [1]. It is characterized by a loss of high-acuity (particularly reading) vision and distortions of the central visual field following the loss of photoreceptor cells in the central retina. Unless effective methods of prevention and treatment are found, the prevalence of AMD is expected to double in the coming decades because of the aging society [2]. AMD has a still unknown etiology; environmental factors as well as different genes have been associated with an increased risk of developing AMD.

Further important causes of macular degenerations include hereditary maculopathies with either autosomal dominant or recessive inheritance that usually occur at early ages, and forms related to drug toxicity, particularly to the intake of chloroquine or clofazimine [3]. Metabolic systemic diseases like diabetes mellitus can also cause a significant disturbance of central vision. Diabetes mellitus affects approximately 20% of American adults over the age of 60 [4], with an increasing incidence in adolescents and younger adults [5]. Many patients develop a diabetic retinopathy, and thus its common complication – diabetic macular edema (DME) – is the most frequent cause of vision loss in patients and a significant public health problem [6].

An increase in understanding particularly of the macula-related pathophysiology may help to establish both an earlier diagnosis and a better treatment of AMD as well as DME and other maculopathies. However, there is a shortage of non-primate animal models that mimic the complex and progressive neuronal and vascular characteristics of macular disorders that are needed to investigate this pathophysiology and to develop specific treatment strategies. Macula-like regions in the sense of cone dominated, avascular regions of maximal visual acuity are found in primates, in some birds, and reptiles [7]. As primate research causes not only ethical concerns but has also economical, time-scale, and statistical problems, the availability of a rodent model would be very valuable.

Although this is strictly speaking not a macula, many mammals possess also a specialized retinal region with an increased density of cones, ganglion and bipolar cells, either in the form of a roughly circular area centralis, or as a horizontal band termed a visual streak [8], [9]. A further commonality is that large retinal vessels skirt these high cell density areas like the macula, and that these regions are vascularized by dense beds of fine capillaries only [10], [11]. In this way, optical degradation of the image is avoided in the areas of highest acuity. Unfortunately, the most commonly used small animal models for vision research, mouse (Fig. 1B) and rat, do not possess a macula or even a retinal region with the aforementioned features. Several mouse models have been developed to understand the underlying mechanisms of the sight-threatening conditions in maculopathies [12], but these models are unable to represent the topographic aspects of the pathophysiology of macular disorders including the vascular component.

In cone-rich non-primate small animal models like e.g. the ground squirrel, studies have so far focused on the neuronal components [13], [14], but little is known about the topography in relation to the vascular pattern. In *Arvicanthis ansorgei*, another model with a relatively high proportion of cones in the retina [15]–[17], no significant specialization of the vascular pattern could be observed. Some laboratory animals with cone-rich retinae like the guinea pig even lack major retinal vessels; the few that are present are small and extend only a short distance from the optic disc (a so-called paurangiotic pattern) [18].

Therefore, the scope of this study was to identify animal models featuring both retinal regions of enhanced cone densities *and* a vascular pattern avoiding these. Another aspect was that they should be easy to breed and to handle in the common housing facilities. For this purpose, we assessed the retinal blood vessel pattern in a number of less commonly used laboratory rodents, which resulted in the identification of three small animal species with specialized retinal regions. Further morphological analyses and assessment of the cone and ganglion cell distributions along with opsin expression revealed topographical differences in retinal composition with distinct similarities to structures of the human macula. In summary, we propose that small animals with specialized retinal regions have the potential to give more insight into the etiology and pathophysiology of human maculopathies and furthermore can be used for the development of effective therapies.

## Results

**Figure 2:**
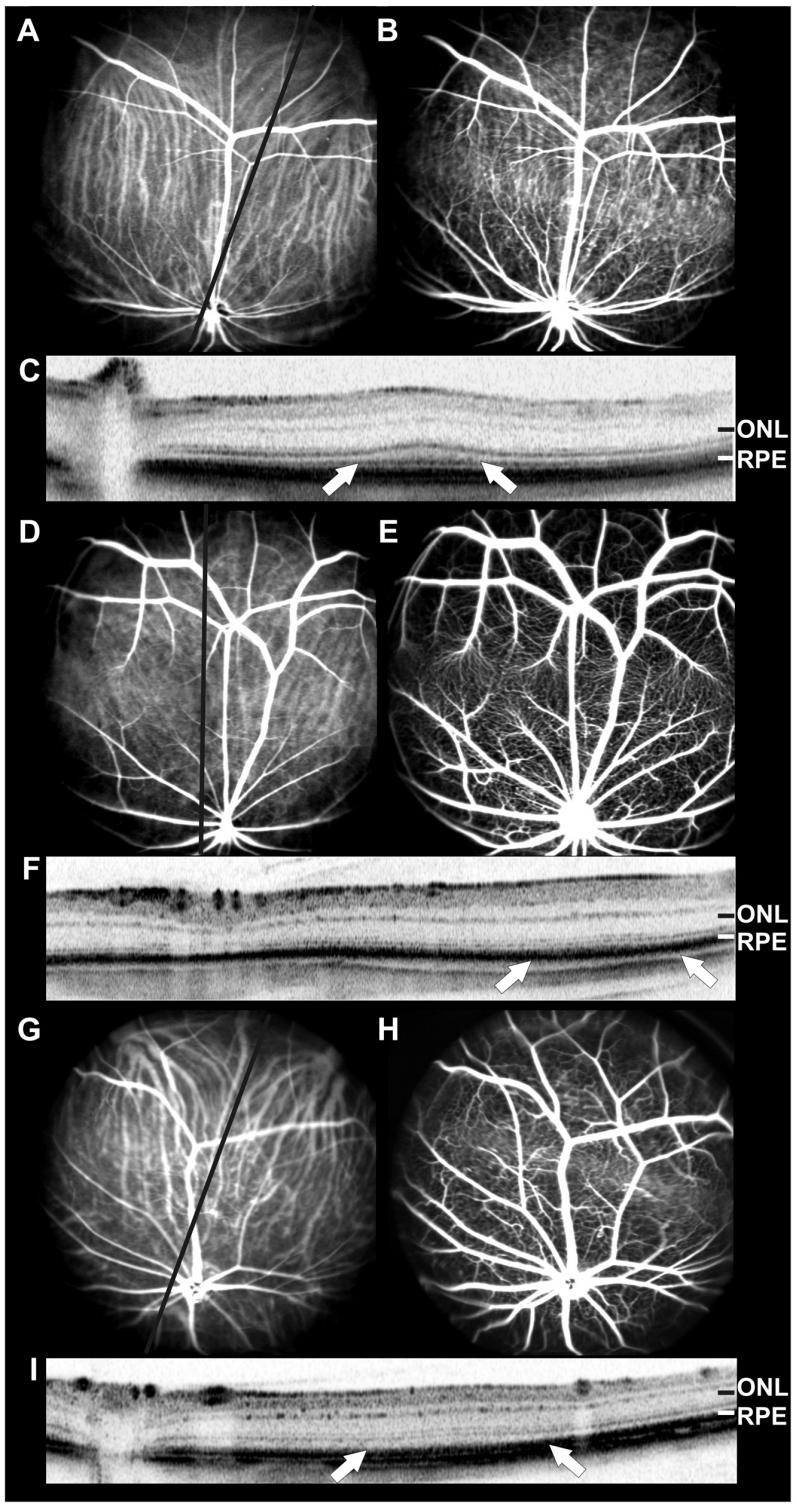
*In vivo* morphological analysis and retinal layering with cSLO angiography and SD-OCT imaging. (**A–C**) Gerbillus perpallidus, (**D–F**) Meriones unguiculatus, and (**G–I**) Phodopus campbelli. cSLO en-face imaging of the vascular pattern using (**A**, **D**, **G**) ICG angiography mode (795 nm with barrier filter at 800 nm) depicts both retinal and choroidal structures. Black line depicts OCT scan direction. (**B**, **E**, **H**) FLA displays large retinal vessels and capillaries. Besides, choroidal vessels are visible in the visual steak region as different retinal layering allows light at λ = 488 nm to better penetrate the RPE/choriocapillary complex. (**C**, **F**, **I**) In vivo cross-sectional SD-OCT images show an increased retinal thickness in the visual streak region, indicating a specialized retinal region (arrows). ONL, outer nuclear layer; RPE, retinal pigment epithilium.

*In vivo* angiography of M. *unguiculatus* using cSLO (Figs. 1C, 2) showed a strikingly different pattern of the retinal blood vessels than in mice (Fig. 1B). The major blood vessels supplying the inner retina divide into several main branches shortly after entering the eye at the optic disc, and then subdivide into a network of capillaries across the retinal surface obviously avoiding a distinct region of the retina. A similar horizontal H-shape of major vessels was also found in *G. perpallidus* and *P. campbelli*. The vessels ramify and narrow into a bed of capillaries as they approach the horizontal sparse vascular band, and except for one artery and one vein only small capillaries invade the region in-between the major vessels. This horizontal sparse vascular band visible in *in vivo* angiography is located dorsal to the optic disc in all three rodent species (Fig. 2). It appears to correspond to a visual streak, very similar to a specialized retinal region like an area centralis or human fovea.

In cSLO angiography (Fig. 2), both dyes used for *in vivo* angiography, fluorescein (FLA) and indocyanine green (ICG), are present in the retinal as well as in the choroidal circulation. Fluorescein angiography provides the most detailed images of retinal capillaries, whereas ICG images depict both retinal and choroidal structures. The degree of visibility of deeper-lying fundus structures depends on the retinal layering [19]. Usually, with fluorescein angiography only the retinal vasculature is visible. However, in the area of the visual streak also choroidal vessels were clearly discernible (Fig. 2B, E, H). This strongly suggested a different retinal layering within the visual streak region. We assessed this question by *in vivo* analysis using SD-OCT imaging. *In vivo* cross-sectional imaging was performed using a commercially available third generation Spectralis HRA+OCT (Heidelberg Engineering, Heidelberg, Germany), which provides high-resolution depth profiles of the retina based on reflectivity of light. This imaging method allows to reliably identify all retinal layers *in vivo* with a resolution comparable to that in histological sections [20]. A significant increase in retinal thickness was found in the visual streak region in comparison to surrounding parts of the retina (Fig. 2C, F, I). Virtual cross-sections displayed identical laminar organization in the inner retina, but in all three species, SD-OCT revealed a clear increase of thickness in the outer retina, particularly in the layer corresponding to the photoreceptor outer segments [21].

**Figure 3:**
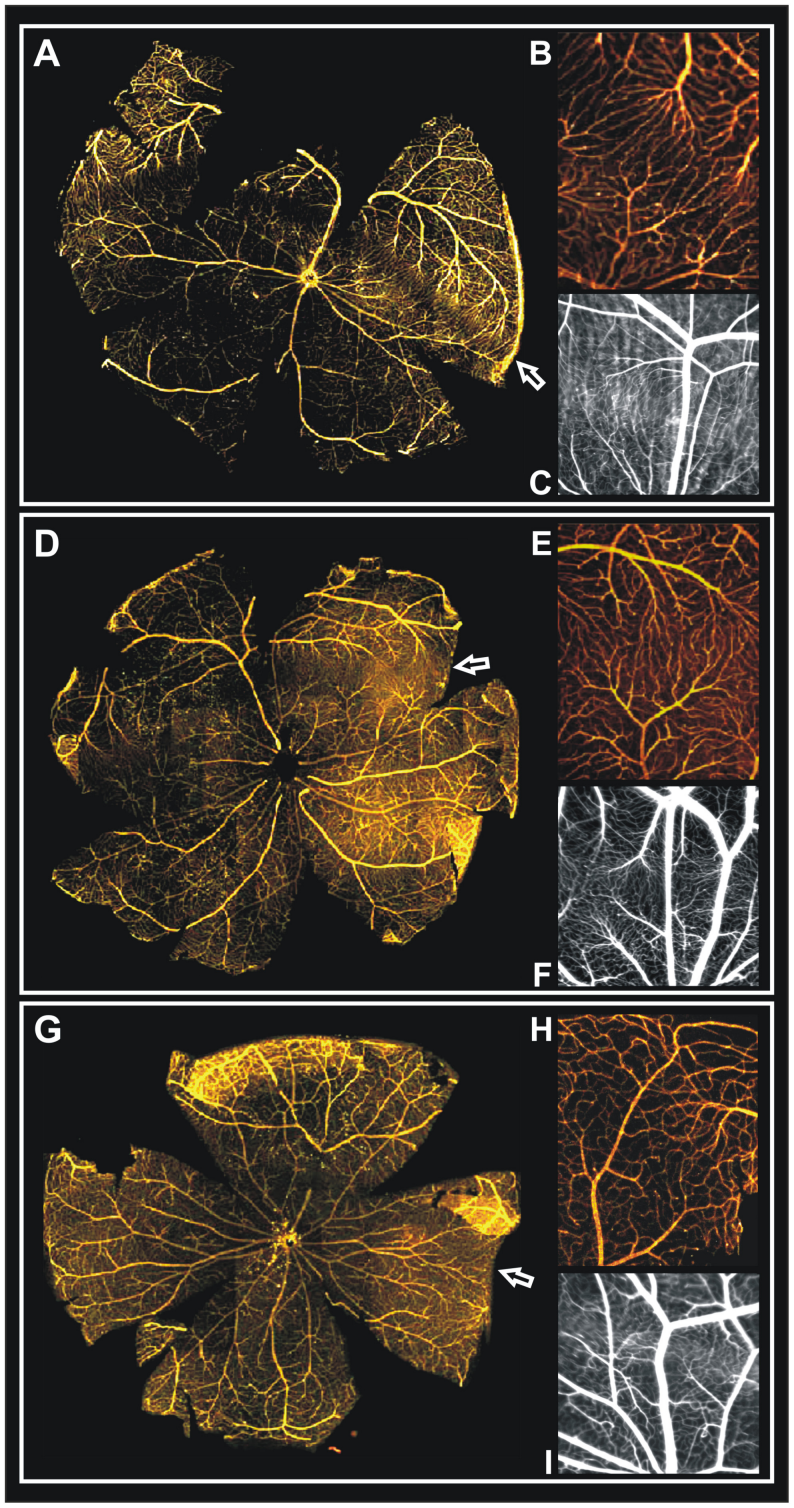
Detailed analysis of the retinal blood vessel distribution *in vivo* using cSLO and *in vitro* using collagen IV staining. (**A–C**) G. perpallidus, (**D–F**) M. unguiculatus, and (**G–I**) P. campbelli. Collagen IV stained retinal wholemounts (**A**, **D**, **G**) illustrate the entire visual streak region. Magnifications of wholemounts (**B**, **E**, **H**) and corresponding in vivo cSLO data (**C**, **F**, **I**) show that retinal vessels ramify and narrow into a sparse capillary net at the border of the respective area.

*In vitro* wholemount stainings allowed visualization and assessment of the three vascular layers in the entire visual streak region and thus complemented *in vivo* cSLO imaging. Collagen IV staining revealed that the specialized vascular patterning was restricted to the superficial vascular plexus within the streak area (Fig. 3). *G. perpallidus* showed a reduced capillary branching in this horizontal streak area. The capillaries traversed the streak area in a vertical pattern. The superficial vessels of *M. unguiculatus* arborized in the horizontal streak region similarly to *G. perpallidus*. In both *G. perpallidus* and *M. unguiculatus*, this specialized vascular patterning was present in a horizontal band that spanned the entire retina. The retinal vasculature of *P. campbelli* showed only slight modifications of the superficial vessels in the streak region, which did not cross the entire retina.

**Figure 4:**
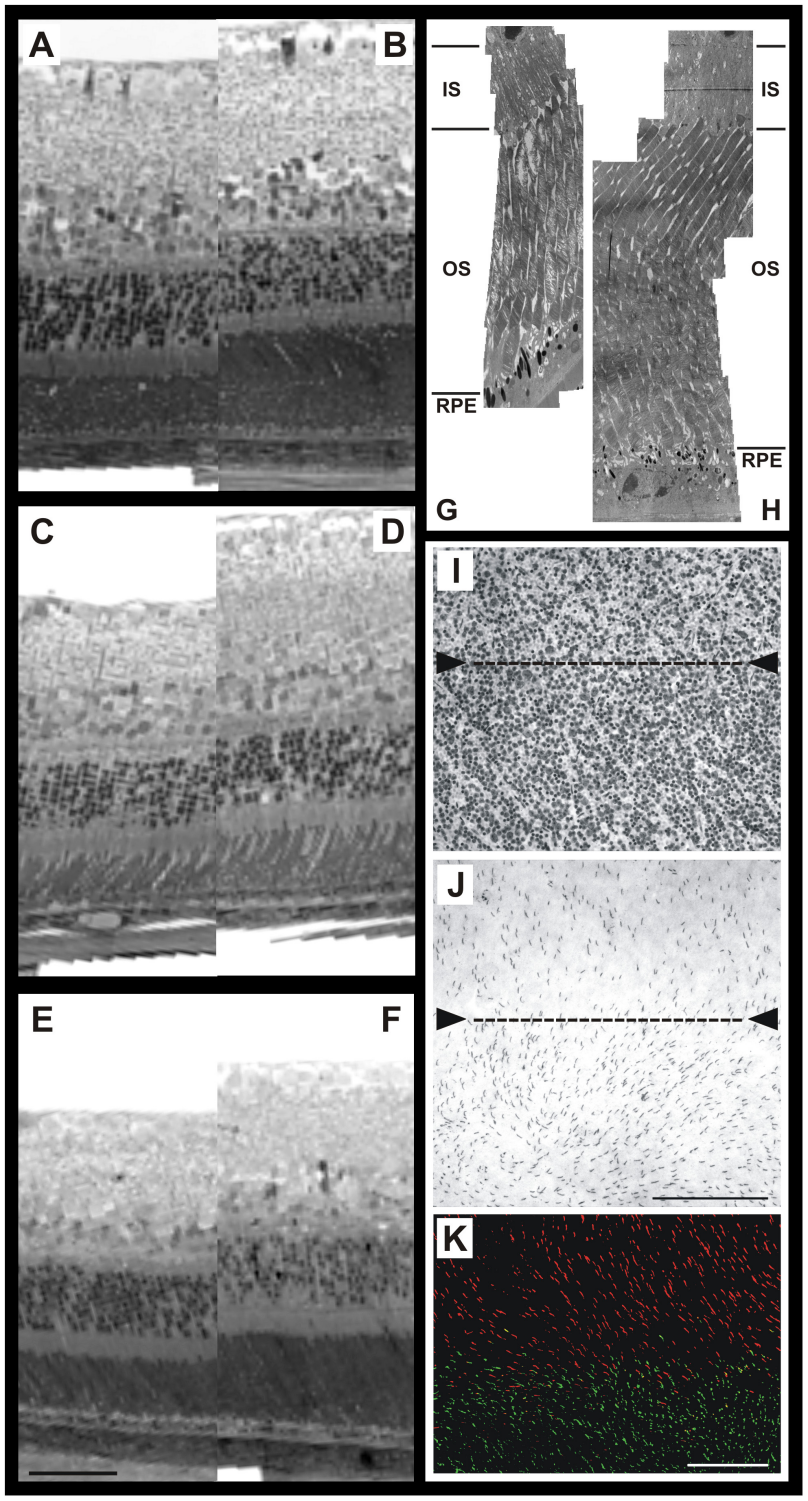
Detailed retinal analysis *in vitro*. (**A**, **B**) G. perpallidus, (**C**, **D**) M. unguiculatus, (E, F) P. campbelli. Methylen blue-stained vertical semithin sections, RPE-aligned, confirming the increase in outer retinal thickness. (**A**, **C**, **E**) outside the visual streak, (**B**, **D**, **F**) within the visual streak region. (**G**, **H**) Representative electron microscopy of G. perpallidus, (**G**) outside the visual streak, (H) within the visual streak region, showing the slanted longer outer segments. (**I**) Ganglion cell layer in the streak region of *G. perpallidus* demonstrated by cresyl violet-staining of a retinal flatmount. Shown is the dorsal edge of the visual streak (arrowheads) with higher neuron densities inside (bottom) than outside (top). (**J**) Cone photoreceptors in a retinal flatmount of *G. perpallidus*, immunolabeled for MWS cone opsin (PAP/DAB). Note the dorsal edge of the streak (arrowheads) with higher cone densities inside (bottom) than outside (top). As the few SWS cones also co-express MWS opsin, the staining shows all cones. (**K**) Cones in a retinal flatmount of *P. campbelli*, double-immunoflourescence labeled for MWS opsin (red) and SWS opsin (green). Shown is the SWS opsin expression edge at the streak, dorsal of the edge only MWS opsin is expressed (top). Scale bar in E = 50 µm for A–F, scale bar in J = 200 µm for I and J, scale bar in K = 100 µm.

The course of the capillary network is very similar in magnifications of both *in vivo* cSLO FLA angiography and *in vitro* collagen IV stained retinal wholemounts (Fig. 3). For further morphological analysis, semithin transverse sections were prepared from Epon-embedded eyes of all three species. Likewise, in these histological sections the streak region could be identified by a thickening of the retina (Fig. 4). Direct comparison of the retinal layering within the streak region (Fig. 4B, D, F) to areas outside (Fig. 4A, C, E) revealed that particularly the layers of the photoreceptor outer segments were substantially enlarged, corroborating the observations from SD-OCT imaging. Electron microscopy further confirmed the significant elongation of the photoreceptor outer segments within the streak region (Fig. 4G, H). Their elongation was roughly estimated to be 1.5 fold. Interestingly, this specialized retinal region generated some difficulties for embedding and cutting. As shown in Fig. 4H the longer outer segments had a slanted orientation in the electron micrographs.

Nissl-stained wholemounts revealed a horizontal visual streak of increased neuron density in the ganglion cell layer (GCL). Among the three species, *G. perpallidus* showed the lowest overall neuron density in the GCL, and a visual streak was very noticeable. Within the visual streak region, a markedly higher neuron density could be detected (Fig. 4I bottom). The increase in neuron density was sharper at the dorsal edge of the streak, ventrally the transition was more gradual. *M. unguiculatus* and *P. campbelli* had overall higher GCL neuron densities across the retina. *M. unguiculatus* also possessed a distinct GCL visual streak with a sharp dorsal edge and a less well-defined ventral edge, whereas *P. campbelli* only showed a very moderate density increase with shallow dorsal and ventral gradients in the streak region.

The labeling of retinal wholemounts for cone opsins revealed inter-species differences. Across the whole retina, *G. perpallidus* had the lowest cone densities among the three species. Most of the cones expressed the middle-to-longwave sensitive (MWS) opsin, and only a few percent the shortwave sensitive (SWS) opsin. In large patches across the retina, SWS opsin was completely absent. All SWS opsin-expressing cones co-expressed the MWS opsin, which is a feature observed in a number of rodents [22]. Cone density had a shallow peak in the streak region, with a distinct drop towards dorsal periphery (Fig. 4J). *M. unguiculatus* had overall higher cone densities. MWS and SWS opsin were expressed across the retina, with MWS cones forming the majority. However, a large proportion of the MWS cones co-expressed small levels of SWS opsin. Highest cone densities were found in ventral retina, and there were no cone specializations associated with the dorsal streak. *P. campbelli* also had high cone densities with maxima in ventral retina. Peculiarly, SWS opsin expression was restricted to the ventral two thirds of the retina. At the streak, SWS opsin expression abruptly ended and dorsal of the streak only MWS cones were present (Fig. 4K). Ventral to the streak, many cones co-expressed both opsins.

**Figure 5:**
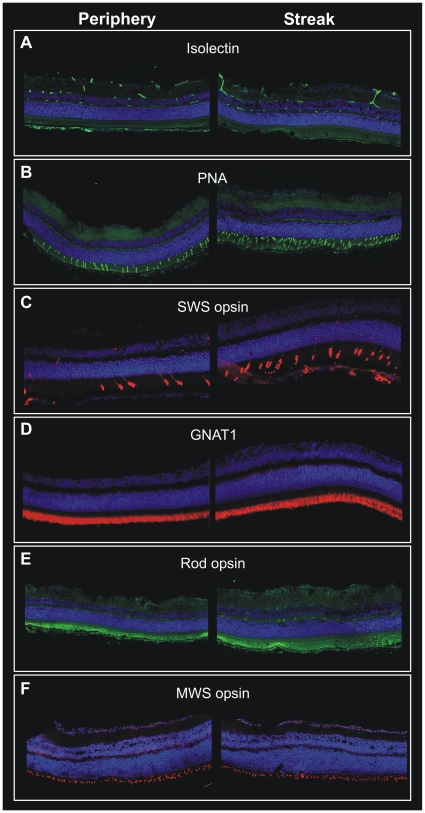
Immunofluorescence labeling of retinal sections revealed distinct differences in cone cell distribution and vessel density. (**A–F**) Areas within the visual streak region are shown in the right panel, areas from dorsal peripheral retina in the left panel. (**A**) Isolectin IB4-FITC staining of G. perpallidus retina reveals the sparse vessel distribution within the streak region; (**B**) Peanut agglutinin staining of P. campbelli retina illustrates increased cone densities within the streak region. (**C**) SWS cone staining of M. unguiculatus retina reveals enhanced SWS cone density within the streak area compared to dorsal periphery. Rods and MWS cones are evenly distributed across the retina as shown by (**D**) rod transducin (GNAT1) staining in M. unguiculatus, (**E**) rod opsin staining, and (F) MWS cone opsin staining in G. perpallidus. (**A–F**) Nuclei were contrasted with DAPI (in blue).

Immunofluorescent stainings of retinal sections revealed distinct differences in cone cell distribution and vessel density (Fig. 5). Isolectin IB4-FITC stained retinal sections of *G. perpallidus* revealed the sparse superficial vascular network within the streak region (Fig. 5A right panel). In comparison to dorsal periphery, peanut agglutinin stained retinae of P. campbelli illustrate increased cone densities within the streak region (Fig. 5B) corresponding to Fig. 4J. More specifically, SWS opsin staining of *M. unguiculatus* retinae revealed enhanced SWS cone density within the streak area (Fig. 5C) compared to the dorsal periphery. Both rods and MWS cones were evenly distributed across the retina as shown by rod transducin (GNAT1) staining in *M. unguiculatus* (Fig. 5D), rod opsin staining (Fig. 5E), and MWS opsin staining in *G. perpallidus* (Fig. 5F). Remarkably, all the antibodies used for immunofluorescent stainings (Fig. 5) stained the same antigens as in mice.

## Discussion

In all three species examined, *Gerbillus perpallidus*, *Meriones unguiculatus*, and *Phodopus campbelli*, a specialized retinal region could be observed, albeit with different characteristics. *M. unguiculatus* had an enhanced SWS cone density within the streak area compared to the dorsal periphery (Fig. 5C). Besides, a pronounced GCL streak and a considerably enlarged IPL were found (Fig. 4C, D). In *P. campbelli*, the peanut agglutinin staining illustrated increased cone densities within the streak compared to the dorsal part of the retina (Fig. 5B). The expression of SWS opsin ends abruptly at the streak, dorsal of this edge there are only MWS cones (Fig. 4K). These properties of *P. campbelli* cones have been described previously [23] and are confirmed by our observations. Interestingly, in the closely related Dzhungarian hamster *Phodopus sungorus* all cones co-express the MWS and SWS opsin, and there is no dorsoventral gradient in SWS opsin expression [24]. This difference deserves further scrutiny. *G. perpallidus* features the most prominent elongation of photoreceptor outer segments (Fig. 4A, B, G, H). Besides, its GCL streak is associated with a relatively high cone density (Fig. 4I).

The analysis of the specialized retinal regions demonstrated similarities to the human macula. In the human retina, the central retinal artery has four main branches, emerging from the optic nerve head and running in a radial fashion curving towards and around the macula. A blood vessel- and capillary-free zone denotes the macular area (Fig. 1). In the animal models described here, a similar characteristic vascular pattern of the superficial vessels and a sparsely vascularized area denoting the specialized retinal region (Fig. 2, Fig. 3, and Fig. 5A) could be observed. The human central retina close to the fovea is considerably thicker than the peripheral retina. This is due to the increased numbers of cones and their associated bipolar and ganglion cells. The enhanced retinal thickness in the animals analyzed here is caused by a relatively high density of cone cells (Fig. 4J, K) as well as a specific elongation of the photoreceptor outer segments in this area (Fig. 4A–H).

The outer nuclear layer (ONL), composed of the cell bodies of the rods and cones has about the same thickness in central and peripheral retina in humans. In the central retina, the cones have oblique axons displacing their cell bodies from their synaptic pedicles in the outer plexiform layer (OPL). These oblique axons with accompanying Müller glial cell processes form a pale-staining fibrous-looking area known as the Henle fiber layer which is absent in the peripheral retina. In the laboratory animals described here, a similar fibrous-looking area could not be identified, however, in histological sections a slightly less ordered alignment of the nuclei in the ONL could be observed in the streak region of all three animals examined, particularly prominent in *M. unguiculatus* (Fig. 4A–F). This different appearance of the ONL may be due to more cone-connected circuits of the neurons and Müller cells spanning the retinal layers within this region.

Furthermore, in humans a remarkable difference between central and peripheral retina can be seen in the relative thicknesses of the inner plexiform layer (IPL), ganglion cell layer (GCL) and nerve fiber layer (NFL). This is again due to the greater numbers and increased packing-density of ganglion cells associated with the cone pathways in the cone-dominated foveal retina as compared to the rod-dominated peripheral retina. The greater number of ganglion cells means more synaptic interaction in a thicker IPL and greater numbers of ganglion cell axons coursing to the optic nerve in the nerve fiber layer. Morphological examinations of the three rodent species showed a similarly enhanced thickness of the GCL and IPL (Fig. 4A–F, I). Particularly in *M. unguiculatus*, the IPL appeared considerably enlarged within the streak region compared to the periphery.

In summary, these models present several features of the human macular region. AMD and DME are retinal degenerative diseases which primarily affect central vision and the macula. It is therefore difficult to model these diseases in mouse and rat, because they both lack a macula. Although mouse models that mimic AMD are available [25]–[30], most of them feature only some of the characteristics of human AMD [31]. Therefore, animal models that mimic the complex and progressive characteristics of AMD are needed to investigate the pathogenesis.

The early stages of AMD are mainly characterized by drusen in the macular area. The pathology of advanced AMD is described to have two forms: the ‘geographic atrophic’ or ‘dry’ and the ‘neovascular/exudative’ or ‘wet’ form, which is often accompanied by hemorrhages, serous exudates, and edema in the neuroretina [32]. Besides wet AMD forms, diabetic macular edema, occurring during progressing diabetic retinopathy, is another important change with leakage of plasma from small blood vessels into the macula. Although diabetic macular edema does not cause total blindness, it frequently leads to severe loss of central vision [5]. The animals described here feature not only a specialized retinal region concerning the retinal layering similar to the human macula; besides, their blood vessel distribution resembles the macular vascular topography. Therefore, these small laboratory rodents might provide a useful tool also for analysis of neovascularization processes in maculopathies. Although there is limited genomic information yet, according to mouse models used so far in macular research, these rodents could equally be used to target genes relevant to AMD, i.e. inflammatory, oxidative stress associated, and metabolic pathway genes [32].

In conclusion, *Gerbillus perpallidus, Meriones unguiculatus*, and *Phodopus campbelli* feature a specialized retinal region resembling key structures of the human macula. Therefore, we suggest that these rodents could be a useful tool to investigate the underlying biological processes and pathomechanisms of macular disorders, and allow scientists and clinicians to test therapeutic options and get answers to questions that would be more difficult and expensive to assess in primate studies or clinical trials.

## Materials and Methods

### Ethics statement

All procedures were performed in accordance with the local ethics committee (Regierungspraesidium Tuebingen), German laws governing the use of experimental animals, and the ARVO statement for the use of animals in ophthalmic and visual research. The Institute of Animal Welfare and the Veterinary Office at the University of Tuebingen insures compliance with all applicable regulations for the use of animals. All examinations are approved by The Institute of Animal Welfare and the Veterinary Office at the University of Tuebingen and the Regierungspraesidium Tuebingen.

### Animals

The present study includes the pallid gerbil, *Gerbillus perpallidus*, the Mongolian Gerbil (Mongolian jird) *Meriones unguiculatus*, and Campbell's Russian dwarf hamster *Phodopus campbelli* [33]. 8 animals of each species were used for the *in vivo* optical imaging approaches, and 10 animals of each species for retinal histology.

### Anesthesia

*Gerbillus perpallidus* and *Meriones unguiculatus* were anesthetized by intraperitoneal injection of a mixture of Fentanyl (0,25 mg/kg), Midazolam (0,75 mg/kg) and Medetomidin (0,075 mg/kg), *Phodopus campbelli* were anesthetized by intraperitoneal injection of a mixture of Fentanyl (0,66 mg/kg), Midazolam (0,66 mg/kg) and Medetomidin (0,33 mg/kg).

### Confocal scanning laser ophthalmoscopy

For en face retinal imaging, we used the commercially available HRA 1 and HRA 2 (Heidelberg Engineering, Heidelberg, Germany) featuring up to two Argon wavelengths (488/514 nm) in the short wavelength range and two infrared diode lasers (HRA 1: 795/830 nm, HRA 2: 785/815 nm) in the long wavelength range. Angiography was performed using s.c.-injected fluorescein dye and a blue (488 nm) laser stimulus with a barrier filter at 500 nm, and indocyanine green using an infrared laser stimulus (795 nm, barrier filter at 800 nm). A detailed protocol for imaging is described elsewhere [19].

### Spectral domain optical coherence tomography (SD-OCT)

SD-OCT imaging was done in the same session as cSLO, i.e. animals remained anaesthetized. Eyes were subjected to SD-OCT using the commercially available Spectralis™ HRA+OCT device from Heidelberg Engineering featuring a broadband superluminescent diode at λ = 870 nm as low coherent light source. Adaptation to the optical properties of small animal eyes was done as described previously [20], [21].

### Retinal wholemount vascular staining

Frozen eyes were fixed in 4% formalin solution for 2 hours. Retina was isolated under a dissection microscope and incubated in permeabilisation/blocking solution (0.5% triton, 1% bovine serum albumine in phosphate buffered saline) for 20 min at room temperature. To stain the retinal vessels the retinae were incubated with rabbit anti-mouse collagen type IV (1∶100 in PBS, Acris, Herford, Germany) at 4°C overnight. After washing with phosphate buffered saline the secondary antibody swine anti-rabbit TRITC (1∶20 in phosphate buffered saline, DAKO, Hamburg, Germany) was incubated for 1 hour at room temperature. Retinal wholemounts were mounted in a 50% glycerol solution on slides and evaluated with a fluorescence microscope (Leica DMRBE, Leica, Wetzlar, Germany).

### Methylen blue-stained semithin sections

Eyes were marked for orientation, then enucleated and immersion fixed in 2.5% glutaraldehyde in 0.1 M cacodylate buffer, pH 7.3, at 4°C overnight. After fixation, cornea and lens were carefully removed and the remaining eyecup was cut through the optic nerve head to separate temporal and nasal parts. Tissue was washed in cacodylate buffer, incubated in osmium tetroxide for 1 h, dehydrated, and embedded in Epon 812 [34]. Sections (0.5 µm) were prepared from the nasal central retina, counterstained with methylene blue and analyzed using a light microscope (Axiovision, Zeiss, Jena, Germany).

### Electron microscopy

Tissue was prepared and fixed as for light microscopy. Sections (50–60 nm) were prepared and contrasted with 4% uranyl acetate in 50% ethanol and 2.6% lead nitrate in 1 M NaOH. Sections were analyzed using a Hitachi 7000 electron microscope (Hitachi, Tokyo, Japan).

### Analysis of cellular distribution in retinal flatmounts

Eyes were marked for orientation, then enucleated, punctured behind the cornea for better penetration of the fixative, and immersion fixed in 4% paraformaldehyde (PFA) in 0.1 M phosphate buffer (PB, pH 7.4) for 2–3 hrs (opsin immunocytochemistry) or several days (Nissl staining of ganglion cells). After thorough washing in PB, the anterior part of the eye was removed, the orientation mark was transferred to the retina, and the retina was carefully isolated from the eyecup.

In free-floating whole retinas, the cone photoreceptors were labeled by the cone opsin-specific antisera JH 492 (directed against the middle-to-longwave sensitive (MWS) opsin, dilution 1∶2000; kindly provided by J. Nathans) and sc-14363 (directed against the shortwave sensitive (SWS) opsin, dilution 1∶500; Santa Cruz Biotechnology Inc.), following previously described protocols [35]. Visualization was by immunofluorescence or by peroxidase-antiperoxidase (PAP) and diaminobenzidine (DAB). Retinas were flattened photoreceptor side up on slides and coverslipped with an aqueous mounting medium.

For staining of the ganglion cell layer, whole retinas were flat-mounted vitreal side up on gelatinized slides and stained on the slide with a 0.1% aqueous solution of cresyl violet following the procedure of Wässle et al. [36]. After dehydration in ethanol and clearing in xylene, the slides were coverslipped with Permount. Cresyl violet stains the Nissl substance, which is most abundant in ganglion cells but also present in some displaced amacrine cells, hence the staining reveals the neuron populations in the ganglion cell layer [9]. Tissue was analyzed with a Zeiss Axioplan 2 microscope. Micrographs were taken with a CCD camera and the Zeiss Axiovision software. Images were adjusted for brightness and contrast in Adobe Photoshop.

### Immunostaining of retinal sections

Eyes were placed in 4% PFA in PB overnight. After removing the cornea and lens, the eyes were fixed further in fresh 4% PFA for 2 hours before being immersed in 30% sucrose in PBS (pH 7.4) at 4°C overnight. The eyes were embedded in tissue freezing medium and frozen in a liquid nitrogen-cooled 2-methylbutane bath. Retinal sections (12 µm thick) were cut and collected on slides. Sections were incubated in a blocking solution (3% normal goat serum and 0.3% Triton X-100 in 0.1 PBS (pH 7.4)) for one hour at room temperature. The sections were incubated with the following primary antibodies at 4°C overnight: anti-SWS cone opsin (sc-14363 (N20), dilution 1∶500, Santa Cruz Biotechnology Inc.), anti-MWS cone opsin (AB5405, dilution 1∶1000, Millipore), anti-rhodopsin (dilution 1∶100; kindly provided by D. Hicks), anti-rod transducin (GNAT1) (sc-389 (K20), dilution 1∶500, Santa Cruz Biotechnology Inc.), anti-isolectin IB4 (L1509, dilution 1∶50, Sigma), PNA (L7381, dilution 1∶250, Sigma), diluted in the blocking solution. As the SWS opsin antiserum was produced in goat, we here used a blocking solution with horse serum and a horse anti-goat secondary antibody. After primary antibody incubation, slides were washed with PBS and incubated with the appropriate secondary antibody in blocking solution for 1 hour at room temperature. Sections were counterstained with DAPI to reveal the nuclear layers. Slides were washed with PBS again and mounted with anti-fade medium (10% Mowiol 4–88 (vol/vol; Calbiochem, San Diego, CA, in 100 mM Tris (pH 8.5), 25% glycerol (wt/vol) and 0.1% 1,4-diazabicyclo [2.2.2] octane (DABCO). Immunofluorescent stainings were analyzed with a digital microscope (Axiovision; Carl Zeiss Meditec, Inc., Dublin, CA).
